# Cinnamic acid abrogates bisphenol A-induced hepatotoxicity via suppression of pro-inflammatory cytokine and modulation of gene expressions of antioxidant enzymes in rats

**DOI:** 10.1016/j.toxrep.2025.101995

**Published:** 2025-03-16

**Authors:** Anne Adebukola Adeyanju, Emmanuel Ayomitide Akinwunmi, Mojisola Esther Karigidi, Olubukola Oyebimpe Agboola, Olusola Olalekan Elekofehinti

**Affiliations:** a Department of Biological Sciences, Faculty of Applied Sciences, KolaDaisi University, Km 18, Oyo Express Road, Ibadan, Oyo, Nigeria; b Department of Biological Sciences, McPherson University, Seriki Sotayo, Ogun, Nigeria; c Department of Biochemistry, Federal University of Technology, Akure, Ondo, Nigeria

**Keywords:** Bisphenol A, Cinnamic acid, Gene expression, Hepatotoxicity, Antioxidants

## Abstract

Bisphenol A (BPA) is regularly used to produce plastic products. Its hepatotoxicity has been unveiled. The effects of cinnamic acid on BPA exposure have not been comprehensively studied, and the key mechanism of action is yet to be unraveled. Rats were allocated into 5 groups. Group 1 (control) was given corn oil. Group 2 received BPA for 14 consecutive days. Group 3 received cinnamic acid at 50 mg/kg in co-administration with BPA while group 4 received cinnamic acid at 100 mg/kg, in co-administration with BPA. Cinnamic acid (CA) only (100 mg/kg) was given to group 5. BPA exposure significantly decreased catalase, glutathione-S-transferase, and superoxide dismutase activities and non-significantly diminished glutathione level. A reduction in the gene expression of catalase accompanied this. Our result showed significant gene elevation at the mRNA level of tumor necrosis factor-α and elevated malondialdehyde by BPA. The significantly elevated alanine transaminase and aspartate transaminase activities in addition to increased levels of total cholesterol, triglycerides, and very low-density lipoprotein with reduced high-density lipoprotein reflected the detrimental effect of BPA in the liver. Our results revealed that cinnamic acid could alleviate the increased pro-inflammatory cytokine level and oxidative stress by downregulating tumor necrosis factor-α gene. The histopathological evaluation confirmed the biochemical results. Hepatic alterations were ameliorated when cinnamic acid was co-administered with BPA. These findings suggest that downregulation of the TNF-α gene induced by cinnamic acid may participate in suppressing the BPA-induced oxidative stress. This offers a new idea to unmask the mechanism underlying cinnamic acid’s interference with BPA-induced hepatic damage.

## Highlights

• The hepatotoxic effect of bisphenol A has been unveiled in a rat model.
• A reduction in the gene expression of antioxidants with elevation of TNF-α at the mRNA level is part of the mechanism underlying BPA-induced hepatotoxicity.
• Cinnamic acid could alleviate the increased pro-inflammatory cytokine level and oxidative stress by downregulating the gene expression of TNF-α.
• Cinnamic acid administration could ameliorate liver dysfunction in BPA-intoxicated rats.

## Graphical Abstract

**Figure:**
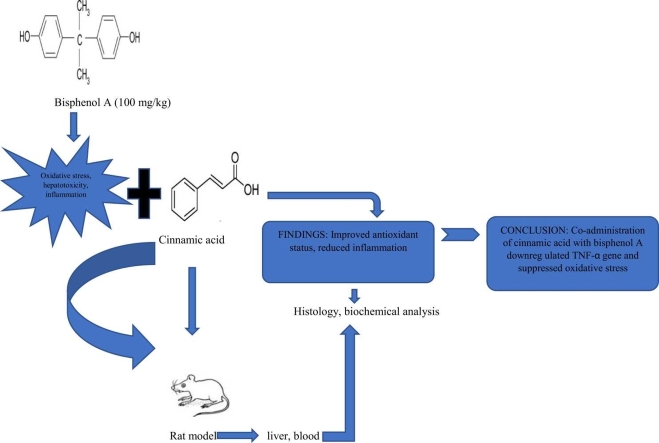

## 1 Introduction

Hepatotoxicity is liver injury (acute or chronic) that occurs after ingestion, inhalation, or transdermal exposure of humans and animals to harsh chemicals including drugs that account for up to 50 % of liver failure [17]. The liver is a vital body part crucial for regulating several physiological functions. Apart from maintaining and regulating homeostasis and its involvement in metabolic processes for growth, resistance to diseases, energy, provision, nutrient delivery, and fat metabolism, the liver is responsible for detoxification (removing toxins) making it highly vulnerable to attacks by harmful chemicals which may eventually result in liver failure [8].

BPA is a chemical component increasingly used worldwide to synthesize polycarbonate plastics for packaging food items and beverages [25]. It induces multi-organ toxicity involving enzyme inhibition, modulation of inflammatory responses, genotoxicity, disruption of neuroendocrine systems, mutagenesis, and carcinogenesis [26]. Kourouma et al., [19] described the induction of apoptosis in liver cells via the induction of reactive oxygen species.

Phytochemicals such as lycopene, luteolin, resveratrol, naringin, and curcumin which are compounds occurring naturally in plants such as tomatoes, green pepper, red grapes, citrus fruits, and turmeric, respectively have been reported to mitigate BPA toxicity by triggering the enzymatic antioxidants to counter BPA-induced oxidative stress [41], [4]. Cinnamic acid (CA) is a class of phenolic acid naturally obtained from the bark of cinnamon. This chemical is a natural aromatic carboxylic acid in plants in the form of whole grains, fruits, and vegetables [37]. Cinnamic acid and its derivatives exhibit antioxidant, antimicrobial, and hepatoprotective properties and have been reported to terminate free radical reactions by supplying electrons to make the radicals stable [30]. It has been proven to have some health-promoting advantages such as antioxidative, anti-inflammatory, and antimicrobial effects. Moreover, cinnamic acid and its derivatives have been studied for their potential to protect against liver damage [40], [44]; breast and colon cancer cell proliferation [14], [33]. Despite the volume of research done to validate the efficacy of cinnamic acid and its derivatives against hepatotoxicity, its protective effect against bisphenol A-induced hepatic injury and its mechanism of action are yet to be unraveled. Therefore, the present study assessed the capacity of cinnamic acid to offer hepatoprotective effects in bisphenol A-induced liver injury in male albino rats.

## 2 Methods

### 2.1 Chemicals and reagents

Ellman's reagent, reduced glutathione, thiobarbituric acid, and trichloroacetic acid were sourced from Sigma. Relevant biochemical kits were obtained from Randox, UK. Cinnamic acid was purchased from Loba Chemie Pvt. Ltd, Mumbai, India. All reagents used were of analytical grade.

### 2.2 The design of experiment

Forty male albino Wistar rats (150–200 g) were obtained and acclimatized for 2 weeks. The animals were then distributed into 5 groups (n = 8), housed in a conventional room in plastic cages. They were fed with a commercial diet and water was given *ad libitum*. Animals were kept at a temperature not exceeding 25°C and a 12 h light/dark during the experiment. Koladaisi University Animal Care and Use Committee approved the protocols and design of the animal experiment. The control group (group I) received corn oil only (1 mg/kg), group II received BPA only (100 mg/kg) [2], [10], group III received BPA in co-administration with 50 mg/kg of cinnamic acid, group IV received BPA 100 mg/kg in co-administration with 100 mg/kg of cinnamic acid, and group V was administered cinnamic acid only (100 mg/kg). The experiment lasted for 14 days [31].

#### 2.2.1 Preparation of liver homogenate

The cervical dislocation method was used to euthanize the animals 24 hours after the experiment's termination. The liver was quickly excised and dissected, cleansed in potassium chloride, blotted and weighed. Subsequently, it was transferred into phosphate buffer and homogenized. The homogenate was centrifuged at 10,000 g for 15 minutes at 4°C to get the post-mitochondrial fraction. It was preserved at 4°C and subsequently utilized for biochemical assay.

#### 2.2.2 Oxidative stress markers

##### 2.2.2.1 Lipid Peroxidation Level

Lipid peroxidation products were identified as slightly modified by [35]. An aliquot (0.4 mL) of the liver post mitochondria fraction was mixed with 1.6 mL of Tris-KCl buffer to which 0.5 mL of 30 % trichloroacetic acid was added. Then 0.5 mL of 0.75 % thiobarbituric acid (TBA) was added and placed in a water bath for 45 minutes at 80 °C. This was then cooled in ice and centrifuged at 3000 *g.* The clear supernatant was collected, and absorbance was measured against a reference blank of distilled water at 532 nm. The malondialdehyde (MDA) level was calculated. Lipid peroxidation in units/mg protein or gram tissue was computed with a molar extinction coefficient of 1.56 × 10^5^ M^−1^cm^−1^.

##### 2.2.2.2 Determination of reduced glutathione (GSH) Level

The method of Beutler et al. [9] was adopted in determining the level of reduced glutathione. The sample (0.2 mL) was added to 1.8 mL of distilled water and 3 mL of precipitating solution was mixed with the sample. The mixture was then centrifuged, and 1 mL of supernatant was added to 4 mL of 0.1 M phosphate buffer. Finally, 0.5 mL of the Ellman’s reagent was added. A blank was prepared with 4 mL of the 0.1 M phosphate buffer, 1 mL of diluted precipitating solution and 0.5 mL of the Ellman’s reagent. Absorbance was taken at 412 nm. GSH concentration was proportional to the absorbance at that wavelength and the estimate was obtained from the GSH standard.

#### 2.2.3 Estimation of antioxidant parameters

##### 2.2.3.1 Measurement of glutathione-S-transferase activity (GST)

It was done with the procedure of [15]. The medium for the estimation was prepared by adding 30 µl of GSH, 150 µl of 1-chloro-2,4-dinitrobenzene, 2.79 mL of phosphate buffer (pH 6.5) and then 30 µl of sample. The reaction was allowed to run for 1 minute each time before the absorbance was read against the blank at 340 nm.

##### 2.2.3.2 Measurement of superoxide dismutase activity

Superoxide dismutase (SOD) activity was determined by Misra and Fridovich [27]. The method is based on the inhibition, by SOD, of the spontaneous autoxidation of adrenaline to adrenochrome at pH 10.2. The reaction was performed at 30 °C in 1 mL of 50 nM sodium carbonate buffer, pH 10.2 containing 0.3 mM adrenaline and 0.1 mM EDTA. One unit of activity is defined as the amount of enzyme required to inhibit the change in absorbance at 480 nm by 50 %.

##### 2.2.3.3 Determination of catalase activity

Catalase activity was estimated using Clairborne's method [11]. The reaction mixture contains 0.09 M H_2_O_2_, 0.1 M phosphate buffer, and then 0.01 mL of sample in a total volume of 3 mL. A double-beam spectrophotometer was used to record the change in absorbance at 240 nm every 30 seconds. The catalase activity was calculated in terms of nmol of hydrogen peroxide consumed/min/mg protein

#### 2.2.4 Liver parameters/lipid profile

Alanine aminotransferase (ALT) and aspartate aminotransferase (AST) activities, and lipid profile were evaluated with randox assay kits.

#### 2.2.5 Protein evaluation

The protein level in tissues was determined by the principle reported by [24]. The absorbance at 578 nm is proportionate to the albumin content in the sample [28].

#### 2.2.6 Histological studies

A segment of rat liver was fixed in a 10 % solution of formalin following the method of Avwioro [7]. The livers were drained in graded alcohol and submerged in paraffin. Fine portions were prepared on glass slides and counter-stained with hematoxylin-eosin for analysis with light microscopes. Then, the slides were assessed by a histopathologist, and photographs were taken.

#### 2.2.7 Gene expression

##### 2.2.7.1 Isolation of total RNA

Total RNA was separated from tissue samples with Quick-RNA MiniPrep™ Kit (Zymo Research). The DNA contaminant was removed following treatment with DNAse I (NEB, Cat: M0303S). The RNA was estimated at 260 nm and the purity was verified at 260 nm and 280 nm using A&E Spectrophotometer (A&E Lab. UK).

##### 2.2.7.2 cDNA conversion

One (1 μg) of DNA-free RNA was utilized to synthesize cDNA by reverse transcriptase reaction with the aid of cDNA synthesis kit based on ProtoScript II first-strand technology (New England BioLabs) in a three-step reaction: 65 °C for 5 min, 42 °C for 1 h, and 80 °C for 5 min [13].

##### 2.2.7.3 PCR amplification and agarose gel electrophoresis

Polymerase chain reaction (PCR) for the amplification of the gene of interest was run with OneTaqR2X Master Mix (NEB) using the following primers (Inqaba Biotec, Hatfield, South Africa). PCR amplification was done in 25 μl volume reaction mixture containing cDNA, primer (see below) and Ready Mix Taq PCR master mix. Under the following conditions: Initial denaturation at 95 ◦C for 5 min, followed by 30 cycles of amplification (denaturation at 95 ◦C for 30 s, annealing for 30 s, and extension at 72 ^◦^C for 60 s) and ending with final extension at 72 ◦C for 10 min. The amplicons were resolved on 1.0 % agarose gel. The GAPDH gene was used to normalize the relative level of expression of each gene, and quantification of band intensity was done using “image J” software [13].

##### 2.2.7.4 Primer sequences

**Superoxide Dismutase**

Forward-AGGGCCTGTCCCATGATGTC

Reverse-AGAAACCCGTTTGCCTCTACTGAA

**Catalase**

Forward-GATGGTAACTGGGACCTTGTG

Reverse-GTGGGTTTCTCTTCTGGCTATG

**Tumor Necrosis Factor-α**

Forward-ACCACGCTCTTCTGTCTACTG

Reverse-CTTGGTGGTTTGCTACGAC

**Nuclear factor erythroid 2-related factor 2**

Forward CACATCCAGACAGACACCAGT

Reverse CTACAAATGGGAATGTCTCTGC

**α-Feto Protein**

Forward: CAGCCAGCAACCATGAAGTG

Reverse: CAACGACAATGGTAGCTACGTTAAA

### 2.3 Statistical analysis

Results were expressed as mean ± standard error of the mean. A one-way analysis of Statistical Package for Social Sciences software for Windows version 16 (SPSS Inc., Redmond, WA, USA) was used for statistical analysis. Post-hoc testing was done for intergroup comparisons using the least significant difference. P < 0.05 represents the level of statistical significance.

## 3 Results

### 3.1 Biochemical assessment

#### 3.1.1 Evaluation of liver function

**Fig. 1:**
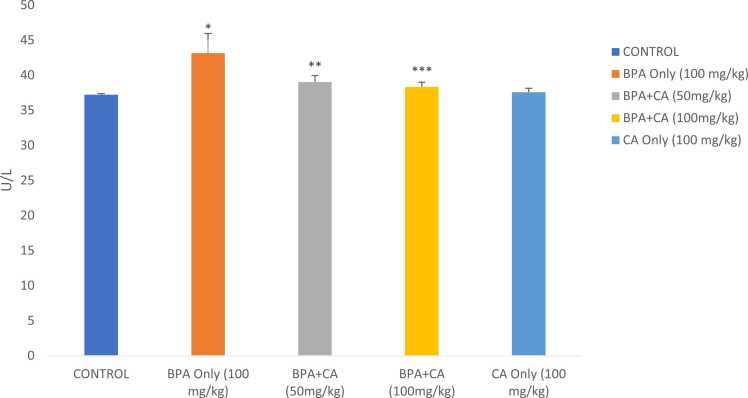
The effect of cinnamic acid on the activities of AST in the liver of rats induced with BPA. Data represent mean ± SD. *P < 0.05 when compared to control. ^**^P < 0.05 when compared to BPA at 50 mg/kg of CA. ^***^P < 0.005 when compared to BPA at 100 mg/kg.

**Fig. 2:**
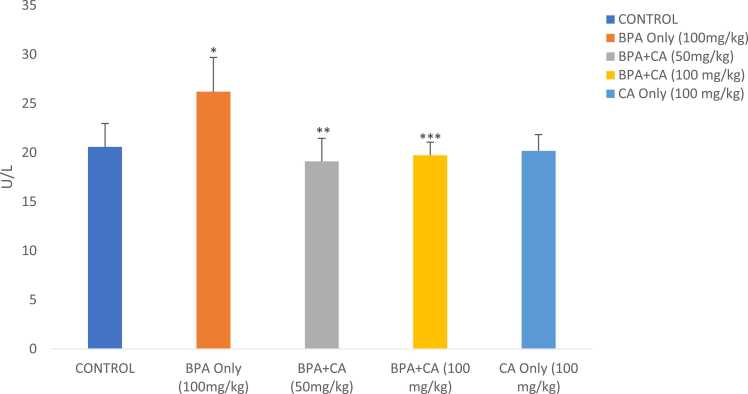
The effect of cinnamic acid on the activities of ALT in the liver of rats induced with BPA. *P < 0.05 when BPA is compared to control. ^**^P < 0.05 compared to BPA at 50 mg/kg of CA and ^***^P < 0.05 compared to BPA at 100 mg/kg of CA.

The effect of cinnamic acid on BPA-induced changes in AST and ALT levels in male albino rats is shown in Fig. 1, Fig. 2. A rise in the activities of ALT and AST was noticed in the BPA group compared to the control (P < 0.05). The combined administration of cinnamic acid with BPA decreased the activities of ALT and AST when compared to the group that received BPA only (P < 0.05). There were no obvious variations in ALT and AST levels of rats that received cinnamic acid only at 100 mg/kg compared to the control group.

#### 3.1.2 Estimation of Antioxidant Parameters

**Fig. 3:**
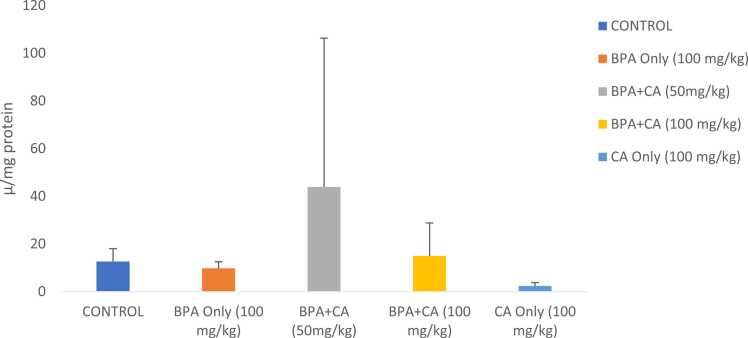
The effect of cinnamic acid on catalase activity in the liver of rats induced with BPA. n = 8 rats per experimental group. Data represent mean ± SD. P > 0.05 in all the groups compared.

**Fig. 4:**
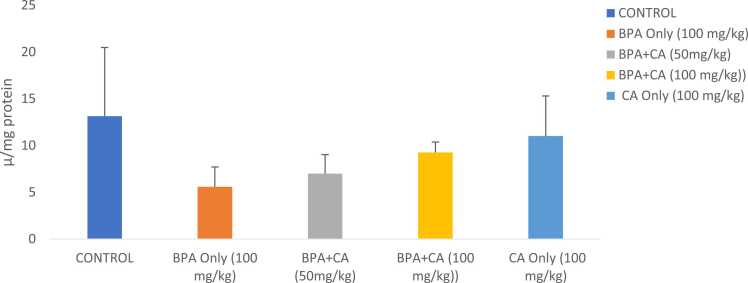
The effect of cinnamic acid on SOD activity in the liver of rats induced with BPA. n = 8 rats per experimental group. Data represent mean ± SD. P > 0.05 in all groups compared.

**Fig. 5:**
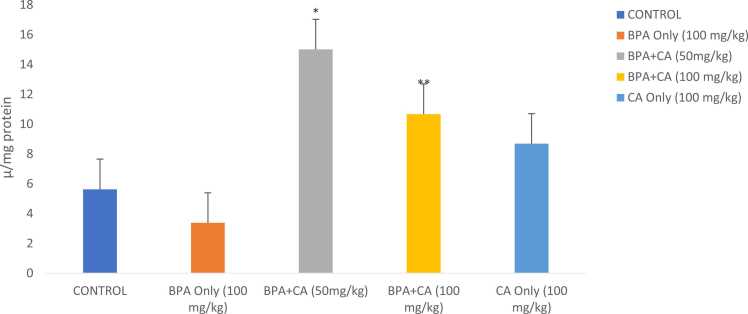
The effect of cinnamic acid on the activities of GST in the liver of rats induced with BPA. n = 8 rats per experimental group. Data represent mean ± SD. *P < 0.05 when compared to BPA at 50 mg/kg of CA and ^**^P < 0.05 when compared to BPA at 100 mg/kg of CA.

Antioxidant activities are shown in Fig. 3, Fig. 4, Fig. 5. Biochemical analysis revealed that rats that were given BPA at 100 mg/kg indicated a considerable decline in catalase, SOD, and GST activities when compared to the control group. However, combined administration of cinnamic acid with BPA for 14 consecutive days significantly modulated catalase, SOD, and GST activities. The 50 mg/kg dose had a noteworthy impact when compared with BPA-treated rats. SOD and GST activities of the group that received cinnamic acid only increased when compared with that of control.

#### 3.1.3 Markers of oxidative stress

**Fig. 6:**
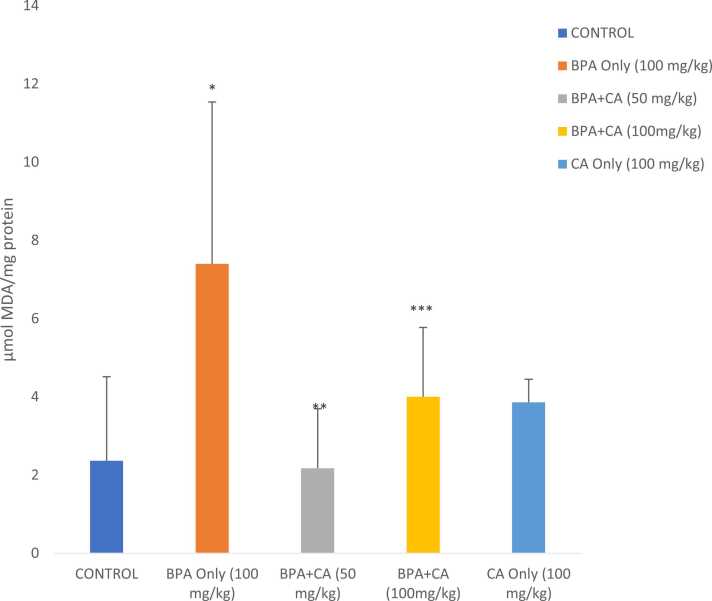
The effect of cinnamic acid on lipid peroxidation level in the liver of rats induced with BPA (100 mg/kg). *P < 0.05 when compared to control. ^**^P < 0.05 when compared to BPA at 50 mg/kg of CA and ^***^P < 0.05 when compared to BPA at 100 mg/kg of CA.

**Fig. 7:**
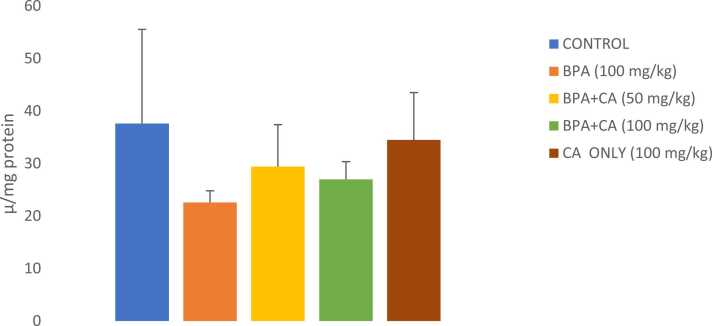
The effect of cinnamic acid on GSH level in the liver of rats induced with BPA. Data represent ± SD. n = 8 rats per experimental group. P > 0.05 in all groups compared.

There was a remarkable surge in MDA level (Fig. 6) in rats exposed to BPA compared to control. However, 14 days of cinnamic acid administration reduced hepatic MDA levels compared to the BPA-only group. The reduction in MDA level was statistically significant. BPA moderately reduced the GSH level below the control level (Fig. 7). The co-administration of cinnamic acid brought the GSH level near the control value (P > 0.05). Cinnamic acid alone (100 mg/kg) caused no considerable effect on liver GSH level compared to the control group.

#### 3.1.4 Evaluation of the lipid profile

**Fig. 8:**
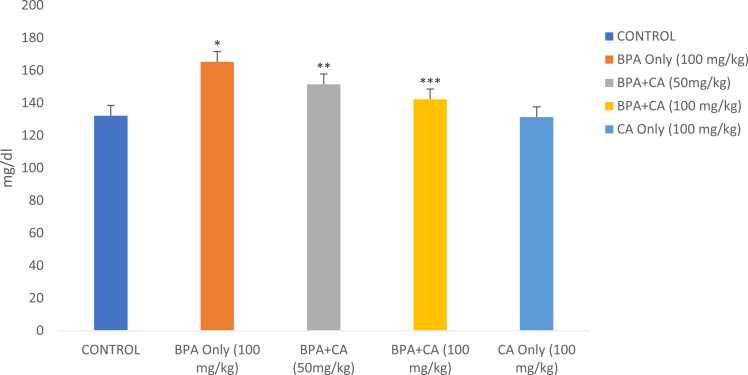
The effect of cinnamic acid on cholesterol level in the liver of rats induced with BPA. *P < 0.05 whenBPA is compared to control. ^**^P < 0.05 when compared to BPA at 50 mg/kg of CA and ^***^P < 0.05 when compared to BPA at 100 mg/kg of CA.

**Fig. 9:**
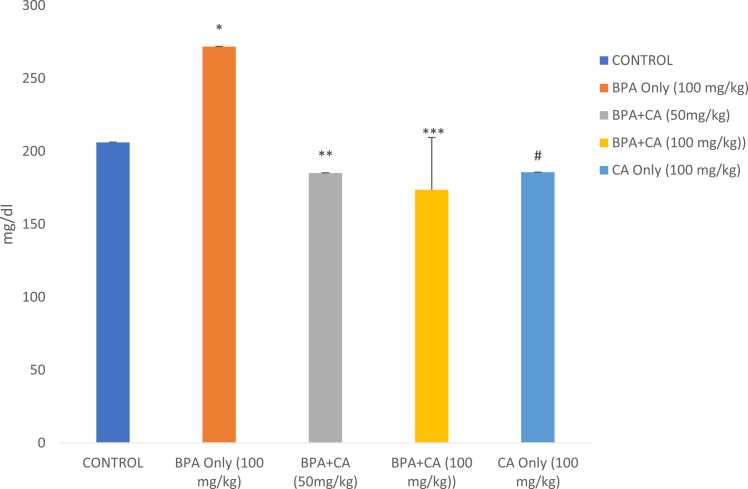
The effect of cinnamic acid on triglyceride level in the liver of rats induced with BPA. Data represent mean ± SD. *P < 0.05 and ^#^P < 0.05 when compared to control. ^**^P < 0.05 when compared to BPA at 50 mg/kg of CA. * **P < 0.05 when compared to BPA at 100 mg/kg.

**Fig. 10:**
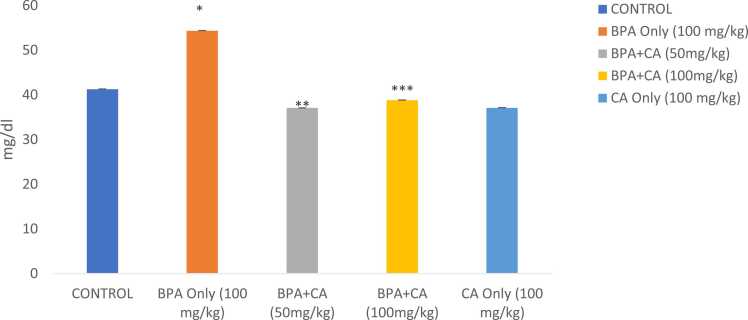
The effect of cinnamic acid on level of VLDL in the liver of rats induced with BPA. n = 8 rats per experimental group. Data represent mean ± SD. *P < 0.05 when compared to control. ^**^P < 0.05 when compared to BPA at 50 mg/kg of CA and ^***^P < 0.05 when compared with 100 mg/kg of CA.

**Fig. 11:**
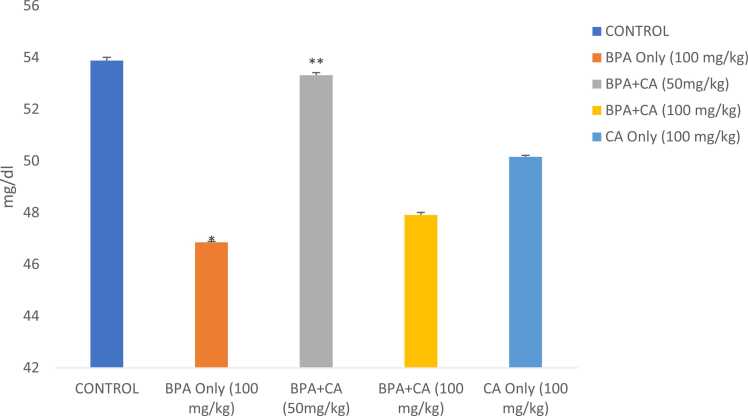
The effect of cinnamic acid on HDL level in the liver of rats induced with BPA. n = 5 rats per experimental group. Data represent mean ± SD. *P < 0.05 when compared to control. ^**^P < 0.05 when compared to BPA at 50 mg/kg of CA.

The effects of BPA on lipid profile are illustrated in Fig. 8, Fig. 9, Fig. 10, Fig. 11. 100 mg/kg of BPA resulted in apparent dyslipidemia expressed by elevated levels of total cholesterol, triglycerides, very-low-density lipoprotein (VLDL), and low high-density lipoprotein (HDL) compared with the corn oil-treated group (P < 0.05). Treatment with 50 and 100 mg/kg of cinnamic acid promoted a high reduction in total cholesterol, triglycerides, VLDL, and an increase in HDL compared with the BPA-only-treated group (P < 0.05). The highest dose of cinnamic acid alone presented no considerable effect on the lipid profile when compared with the control.

#### 3.1.5 Histopathological study

**Fig. 12:**
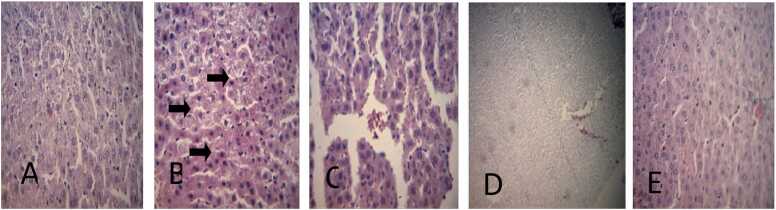
The photomicrographs of histopathological examination (×400) of liver of rats treated with BPA and CA: (A) group treated with corn oil shows no visible lesions (B) group treated with BPA only shows a mild focally extensive hydropic degeneration of hepatocytes (C) group treated with BPA (100 mg/kg) and CA (50 mg/kg) shows no visible lesions (D) group treated with BPA and CA (100 mg/kg each) shows no visible lesions (E) group treated with CA only (100 mg/kg) shows no visible lesions.

The histopathological examination of the liver of rats treated with BPA and cinnamic acid is shown (Fig. 12). Rats in the control group displayed a normal histological appearance while the liver sections from the BPA-treated group showed a mild focally extensive hydropic degeneration of hepatocytes. The BPA-treated groups co-administered with cinnamic acid at 50 and 100 mg/kg notably improved the above histopathological findings compared to the BPA-treated group.

#### 3.1.6 Gene expression

**Fig. 13:**
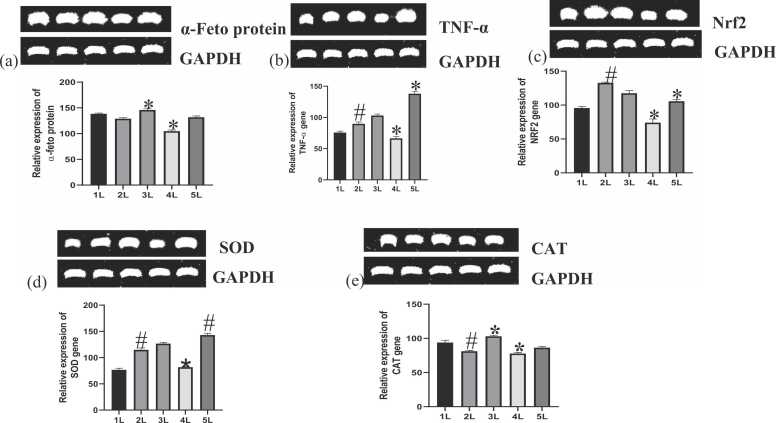
Effect of BPA and cinnamic acid on the gene expression of (a) α-feto protein (b)TNF-α (c) Nrf2 (d) SOD (e) CAT. *Significantly different from BPA-administered group (p < 0.05). ^#^Significantly different from the control group (p < 0.05).

The genes of α-feto protein, tumor necrosis factor α (TNF-α), Nuclear factor erythroid 2-related factor 2 (Nrf2), SOD, and catalase were investigated in the liver as depicted in Fig. 13 (a-e). In Fig. 13(a), α-feto protein gene expression was slightly lowered as compared to the control (p > 0.05) following the administration of BPA. Co-administration of cinnamic acid at 50 mg/kg remarkably upregulated the expression of α-feto protein (P < 0.05). At 100 mg/kg, there was a remarkable depletion in expression compared to the BPA-treated group. The level of α-feto protein gene in rats that received 100 mg/kg of cinnamic acid was not different from the control rats.

In Fig. 13(b), there occurred a notable rise in the expression of TNF-α gene in the BPA-administered group compared to the control (P < 0.05). However, co-administration with cinnamic acid (lower dose) led to a rise in TNF-α gene expression but a significant decrease in expression was observed at higher dose. In group 5, the administration of cinnamic acid alone triggered an overexpression of TNF-α gene.

As demonstrated in Fig. 13(c), administration of BPA significantly upregulated Nrf2 expression compared to the control group. Co-administration of cinnamic acid in groups 3 and 4 showed a decline in the expression of Nrf2 gene. In group 5, cinnamic acid showed a similar level of Nrf2 expression compared to control.

In Fig. 13(d), SOD expression was significantly upregulated by the administration of BPA compared to control. Co-administration with cinnamic acid in group 3 increased the expression of SOD gene but was downregulated considerably by 100 mg/kg co-administration of cinnamic acid. Cinnamic acid in group 5 significantly improved the expression of SOD gene.

BPA administration significantly lowered the expression of catalase gene compared to control group (Fig. 13e). However, co-administration with cinnamic acid at 50 mg/kg remarkably upregulated the expression of catalase while at a higher dose, the gene was significantly downregulated. Cinnamic acid alone showed catalase expression like the control group.

## 4 Discussion

The liver is involved in the biochemical and signaling pathways linked with homeostasis and detoxifies xenobiotics. BPA is an abundant chemical compound in the environment and its long-term exposure can generate oxidative stress and risks to the liver and human health [46]. Oxidative stress is a useful index of liver injury, which expresses a disproportion in the redox system [5]. Earlier studies have revealed the influence of BPA on human health and its toxic effects have been ascribed to increased oxidative stress [3].

The liver has an intrinsic antioxidant defense system for preventing cellular impairment. However, the antioxidant activities of liver catalase, SOD, and GST diminished with 100 mg/kg of BPA received by rats for 14 consecutive days. At the same time, hepatic MDA level significantly rose. SOD dismutates superoxide anion radical into hydrogen peroxide, which is broken down by catalase using GSH. The suppression in catalase activity may lead to failure to degrade hydrogen peroxide formed after BPA exposure [38]. Consequently, hydrogen peroxide may build up and initiate lipid peroxidation [32]. BPA produces free radicals such as semiquinone intermediates that can react with glutathione to generate glutathione conjugates and enhance oxidative stress level [12]. In the present study, depletion of GSH level by BPA could alter redox homeostasis and disrupt antioxidant defense, which explains the oxidative stress observed in liver cells. The deficiency in GSH content may be accountable for the diminished GST activity observed after BPA administration. GST is a detoxifying enzyme that offers protection by conjugating GSH to electrophilic substrates, producing less reactive and more soluble compounds [39]. A significant reduction in its activity would result in its inability to detoxify this compound. The results from this study are congruent with the report of Acaroz et al. [1] who demonstrated a decline in activities of SOD and CAT and reduced glutathione level in rats subjected to bisphenol A at different doses. Overall, BPA seems to react with oxygen radicals and decomposes them into reactive metabolites with strong oxidant capacity. Consequently, these metabolites promote the formation of reactive oxygen species which may potentiate the separation of peptide chains and the cross-linking of amino acids in enzymes, loss of antioxidant enzymes activities, and rise in hydrogen peroxide and lipid peroxidation product levels [42], [18]. These may explain the process of BPA-induced oxidative damage.

In this study, BPA-induced hepatotoxicity at 100 mg/kg in rats is corroborated by Patrick *et al*., (2024). This is evident in the changes in ALT and AST studied. The induction of oxidative stress and lipid peroxidation by BPA can contribute to cytosolic leakage of these enzymes into the bloodstream causing hepatic impairment and interfering with the integrity of cellular membranes [36]. Modifications of the status of these liver enzymes can signify liver injury or interruption of bile flow [6].

Also, BPA-induced toxicity is associated with deterioration in lipid profile. The significant rise in total cholesterol, triglycerides, and LDL levels with a concomitant decrease in HDL observed in rats exposed to BPA has been reported at acute levels [20] and could develop into hyperlipidemia. Liu et al. [23] and Li et al. [21] have reported evidence of BPA-induced hyperlipidemia. The BPA-induced lipid accumulation may be triggered by the exaggerated expression of the transcription factor, sterol regulatory element binding protein-1, which elevates the activity of the enzymes that catalyze lipogenesis [22].

Findings revealed an extensive hydropic degeneration of hepatocytes which might indicate acute liver injury due to BPA treatment. Taken together, the biochemical assessment and histopathology results suggest that BPA exposure induced liver damage in rats.

Co-administration with cinnamic acid for 14 days signaled that the deleterious effects of BPA on the rat’s liver were ameliorated as shown in the improvement in the inhibition of oxidative stress and efficiency of the antioxidant enzymes. This agrees with prior studies revealing that cinnamic acid can reduce oxidative stress in pathological conditions [16]. Amelioration of oxidative stress by cinnamic acid can be ascribed to its reactive oxygen species scavenging activity [34] and its capacity to upregulate the mRNA expression of antioxidant enzymes.

The gene expression of α-feto protein, a biomarker used for assessing hepatocellular toxicity was examined in this study. α-feto protein gene expression was a bit lower in the BPA group compared to the control group. This shows that at 100 mg/kg, BPA administration did not adversely upregulate the expression of α-feto protein. Surprisingly, co-administration with 50 mg/kg of cinnamic acid triggered a significant upregulation of the gene. There was a remarkable reduction at a higher dose of 100 mg/kg cinnamic acid compared to the BPA-treated group.

Tumor Necrosis Factor (TNF)-α is an inflammatory cytokine associated with liver inflammation. Its elevated level is a primary feature of an inflammatory response. In this study, the gene expression of TNF-α was significantly increased by BPA which is similar to the result recently published by Nagarajan et al. [29] which showed significant hepatic gene elevation at the mRNA level of TNF-α by BPA. Data generated revealed that the increased pro-inflammatory cytokine level could be alleviated by CA treatment. Cinnamic acid administered at 100 mg/kg significantly ameliorated this effect by downregulating the TNF-α gene.

Nrf2 is considered an important regulator of the antioxidant defense system. It reduces and hinders the progression of tissue inflammation and mops up ROS generation. The administration of BPA triggered a significant upregulation of Nrf2 gene compared to the group exposed to corn oil. Meanwhile, co-administration with cinnamic acid at both concentrations investigated reduced the expression of Nrf2 gene. This indicates that BPA at 100 mg/kg did not adversely affect the expression of hepatic Nrf2 at mRNA level.

The level of gene expression of enzymatic antioxidants SOD and CAT were measured to determine the effect of BPA and cinnamic acid on the antioxidant status of rat liver. Gene expression of SOD at 100 mg/kg of BPA administered was significantly higher than the control in this study. An earlier study by [23] corroborates this as the administration of BPA at a concentration lower than 270 mg/kg presented no reduction in SOD activity and this might be considered an adaptive response at an early stage of inducing toxicity. Co-administration of cinnamic acid at 50 mg/kg precipitated a surge in the expression of SOD. There was a significant reduction in the expression of CAT gene by BPA. However, co-administration with 50 mg/kg of cinnamic acid increased CAT gene expression. This shows that cinnamic acid can boost the antioxidant status of the liver of BPA-administered rats. It aligns with previous studies that reported cinnamic acid's ability to improve rats' antioxidant status after exposure to a hepatotoxic agent [30], [45].

The accumulation of hepatic MDA, decrease in antioxidant enzymatic activities and their gene expression coincide with up-regulation of TNF-α by BPA in the liver, indicating the involvement of this cytokine in oxidative stress and liver malfunctioning. Therefore, it is proposed that BPA-induced pro-inflammatory cytokine accompanied by the production of oxidative stress, is the fundamental mechanism instrumental to BPA-induced hepatotoxicity. This hypothesis connects with the results of Wahby et al. [43] who attributed the hepatic damage caused by BPA to the induction of TNF-α, advanced oxidative stress, and a decline in antioxidant activities. The mechanism of cinnamic acid protection against BPA-induced hepatotoxicity could be mediated by reinforcing the antioxidative system and anti-inflammatory activities.

## 5 Conclusion

This study's results indicated, for the first time, the effectiveness of cinnamic acid administration in ameliorating liver dysfunction in BPA-intoxicated rats.

## Funding sources

This research did not receive a specific grant from funding agencies in the public, commercial, or non-profit sectors.

## Author statement

The authors declare that this manuscript is original, has not been published before, and is not currently being considered for publication elsewhere. All authors who contributed to this manuscript have been listed accordingly. We affirm that they have read and approved the manuscript. Also, all of us have approved the arrangement of the authors’ names as listed in the manuscript and the corresponding author.

## CRediT authorship contribution statement

**Karigidi Mojisola Esther:** Writing – original draft, Methodology, Conceptualization. **Akinwunmi Emmanuel Ayomitide:** Writing – original draft, Project administration, Methodology, Investigation, Funding acquisition. **Elekofehinti Olusola Olalekan:** Writing – original draft, Methodology. **Agboola Olubukola Oyebimpe:** Writing – original draft, Methodology, Conceptualization. **Adeyanju Anne Adebukola:** Writing – review & editing, Writing – original draft, Methodology, Investigation, Funding acquisition, Formal analysis, Conceptualization.

## Declaration of Competing Interest

The authors declare that they have no known competing financial interests or personal relationships that could have appeared to influence the work reported in this paper

## Data Availability

Data will be made available on request.
